# Effect of Artificial Intelligence-based Health Education Accurately Linking System (AI-HEALS) for Type 2 diabetes self-management: protocol for a mixed-methods study

**DOI:** 10.1186/s12889-023-16066-z

**Published:** 2023-07-11

**Authors:** Yibo Wu, Hewei Min, Mingzi Li, Yuhui Shi, Aijuan Ma, Yumei Han, Yadi Gan, Xiaohui Guo, Xinying Sun

**Affiliations:** 1 Department of Social Medicine and Health Education, School of Public Health, Peking University, Beijing, China; 2 School of Nursing, Peking University, Beijing, China; 3 Beijing Center for Disease Control and Prevention, Beijing, China; 4 Beijing Medical Examination Center, Beijing, China; 5 Daxing District Center for Disease Control and Prevention of Beijing, Beijing, China; 6 Peking University First Hospital, Beijing, China

**Keywords:** Type 2 diabetes, Artificial intelligence, Intelligent question and answering, Mobile health, Mixed-methods study

## Abstract

**Background:**

Patients with type 2 diabetes (T2DM) have an increasing need for personalized and Precise management as medical technology advances. Artificial intelligence (AI) technologies on mobile devices are being developed gradually in a variety of healthcare fields. As an AI field, knowledge graph (KG) is being developed to extract and store structured knowledge from massive data sets. It has great prospects for T2DM medical information retrieval, clinical decision-making, and individual intelligent question and answering (QA), but has yet to be thoroughly researched in T2DM intervention. Therefore, we designed an artificial intelligence-based health education accurately linking system (AI-HEALS) to evaluate if the AI-HEALS-based intervention could help patients with T2DM improve their self-management abilities and blood glucose control in primary healthcare.

**Methods:**

This is a nested mixed-method study that includes a community-based cluster-randomized control trial and personal in-depth interviews. Individuals with T2DM between the ages of 18 and 75 will be recruited from 40-45 community health centers in Beijing, China. Participants will either receive standard diabetes primary care (SDPC) (control, 3 months) or SDPC plus AI-HEALS online health education program (intervention, 3 months). The AI-HEALS runs in the WeChat service platform, which includes a KBQA, a system of physiological indicators and lifestyle recording and monitoring, medication and blood glucose monitoring reminders, and automated, personalized message sending. Data on sociodemography, medical examination, blood glucose, and self-management behavior will be collected at baseline, as well as 1,3,6,12, and 18 months later. The primary outcome is to reduce HbA1c levels. Secondary outcomes include changes in self-management behavior, social cognition, psychology, T2DM skills, and health literacy. Furthermore, the cost-effectiveness of the AI-HEALS-based intervention will be evaluated.

**Discussion:**

KBQA system is an innovative and cost-effective technology for health education and promotion for T2DM patients, but it is not yet widely used in the T2DM interventions. This trial will provide evidence on the efficacy of AI and mHealth-based personalized interventions in primary care for improving T2DM outcomes and self-management behaviors.

**Trial registration:**

Biomedical Ethics Committee of Peking University: IRB00001052-22,058, 2022/06/06; Clinical Trials: ChiCTR2300068952, 02/03/2023.

**Supplementary Information:**

The online version contains supplementary material available at https://doi.org/10.1186/s12889-023-16066-z.

## Background

Diabetes mellitus is a common chronic disease with a high prevalence and mortality rate. The number of people with diabetes aged 20–79 years is estimated to be at 537 million in 2021, which is still rising and is expected to reach to 643 million by 2030, and 783 million by 2045. There are 140.9 million adults with diabetes in China, with a prevalence of 11.9%, causing a great health and economic burden [1]. However, diabetes control and treatment in China is inadequate, indicating the need for effective and innovative methods to improve diabetes prevention and treatment.

Diabetes self-management is one of the cornerstones of diabetes control, and it entails lifestyle changes such as diet and physical activity (PA), blood glucose monitoring, and medication administration on a regular basis. The body of evidence has shown the benefits of diabetes self-management on metabolic control, outcome improvements and reduced risk of all-cause mortality, which is recommended by international consensus [2]. Dietary management involves healthy dietary patterns to achieve and maintain optimal blood glucose, lipid, and blood pressure levels [3–6], such as Mediterranean diet [7, 8], the Dietary Approaches to Stop Hypertension (DASH) diet [9–11], the low calorie energy deficit diet [12–14], the low carbohydrate diet [15–17], vegan and vegetarian diets [18, 19], intermittent fasting and macrobiotic diets [20], and the low glycaemic index or glycaemic load dietary patterns [21–24]. In addition, a sedentary lifestyle, which parallels the global obesity epidemic, is also a crucial risk factor of T2DM [25, 26]. Physical activity, such as various leisure time PA and occupational PA, has been shown to reduce the risk of T2DM, which may partly be mediated by reduced adiposity [27, 28]. Several studies have demonstrated that PA improves insulin sensitivity, glycaemic control, lipid profile, and blood pressure [29–31], with benefits lasting from 2 to 72 h after any PA [32]. Furthermore, regular self-monitoring of blood glucose (SMBG) and medication-taking are frequently considered in the context of diabetes self-management education and support [33]. Diabetes self-management education aims to improve diabetes-related health literacy, self-efficacy, and self-management abilities in a variety of contexts and forms for patients with diverse backgrounds, experiences, and clinical information. To improve the effectiveness, accessibility, acceptability, and cost-effectiveness of diabetes education, a variety of new and innovative technologies need to be applied, and they are more effective when used together for better metabolic control and outcomes [34–36].

Mobile health (mHealth) is increasingly being implemented into health care practice, revealing special benefits in providing rapid, abundant, and tailored medical information to various groups of individuals, with lower health care costs and removed time and place restrictions [36–38]. A number of randomized controlled trials have shown the efficacy of applying mHealth technologies for lifestyle change and HbA1c improvement in T2DM [39, 40], such as the Norwegian Randomized Controlled Trial RENEWING HEALTH [41], MyPlan 2.0 intervention (a self-regulation–based electronic and mHealth project) in two randomized controlled trials [42], a digital medicine offering (DMO) in a cluster-randomized pilot trial [43], and a fully automated Web-based program in the ANODE study [44]. However, despite the availability of a wide range of diabetes-specific mHealth apps available, more detailed evidence regarding their clinical usefulness is still required [45].

Artificial intelligence, defined as “a field of science and engineering concerned with the computational understanding of what is commonly called intelligent behavior and with the creation of artifacts that exhibit such behavior” [46], is being developed and applied in a variety of healthcare fields, especially decision support and knowledge acquisition. There are diverse intelligent algorithms and techniques in AI, such as machine learning (ML), natural language processing (NLP), robotics, fuzzy logic (FL), expert systems (ES), knowledge base (KB), and a combination of two or more methods [46]. To tackle clinical difficulties in T2DM, a large amount of data collecting, analysis, and application of knowledge is required. Therefore, an increasing number of AI-based projects for T2DM diagnosis [47–50], clinical decision-making [51–53], and disease probability or outcome prediction [46, 54–57] have been developed. knowledge graph is a research field of AI that is graph that includes entities, properties, and relations between entities, which is usually stored in the form of inter-connecter triples [58]. KG-based technologies are being developed to extract structured knowledge from massive data, which is considered as a crucial component for the development of AI in conjunction with big data and deep learning [58], especially in the medical domain [59]. Potential applications of KG technology in T2DM include medical information retrieval, medical knowledge intelligent question and answering (QA), diagnosis, and treatment. Knowledge-based question answering (KBQA) is a type of QA processing that uses a knowledge database to provide fast and accurate answers to questions written in natural language expressions by users [60]. It has been applied in disease diagnosis and treatment recommendation [61–63]. However, KBQA in T2DM is a research area that has yet to be fully investigated. Chang, et al. developed DiaKG, the first high-quality Chinese dataset for the diabetes knowledge graph, with 22,050 entities and 6,890 relations about clinical research, drug usage, clinical cases, diagnosis, and treatment methods of T2DM [59]. Chen, et al. created a KBQA-based personalized food recommendation framework based on FoodKG and American Diabetes Association (ADA) lifestyle guidelines that outperformed non-personalized counterparts in terms of recommending more relevant and healthier recipes [64]. Due to a lack of high-quality T2DM annotated corpora and KG, few studies used KG as a T2DM intervention to assess its effectiveness on self-management and clinical outcomes.

In summary, given the great potential of patient-oriented KBQA technology for diabetes prevention and control in primary healthcare, the primary goal of this study is to develop a personalized, sustainable and cost-effective health education intervention program, AI-HEALS, to improve the outcome and self-management skills of people with T2DM.

The specific objectives are:

- 1. Evaluate the impact of the AI-HEALS-based intervention program on blood glucose control of T2DM patients.
- 2. Determine the efficacy of the AI-HEALS-based intervention program in improving self-management abilities among T2DM people:
-
  - 2.1 The effectiveness of a healthy diet;
  - 2.2 The effectiveness of moderate PA;
  - 2.3 The effectiveness of regular medication administration;
  - 2.4 The effectiveness of regular blood glucose monitoring.
  - 3. Compare the acceptability and cost-effectiveness of AI-HEALS-based intervention program in T2DM primary healthcare.

## Methods

### Study design

**Fig. 1:**
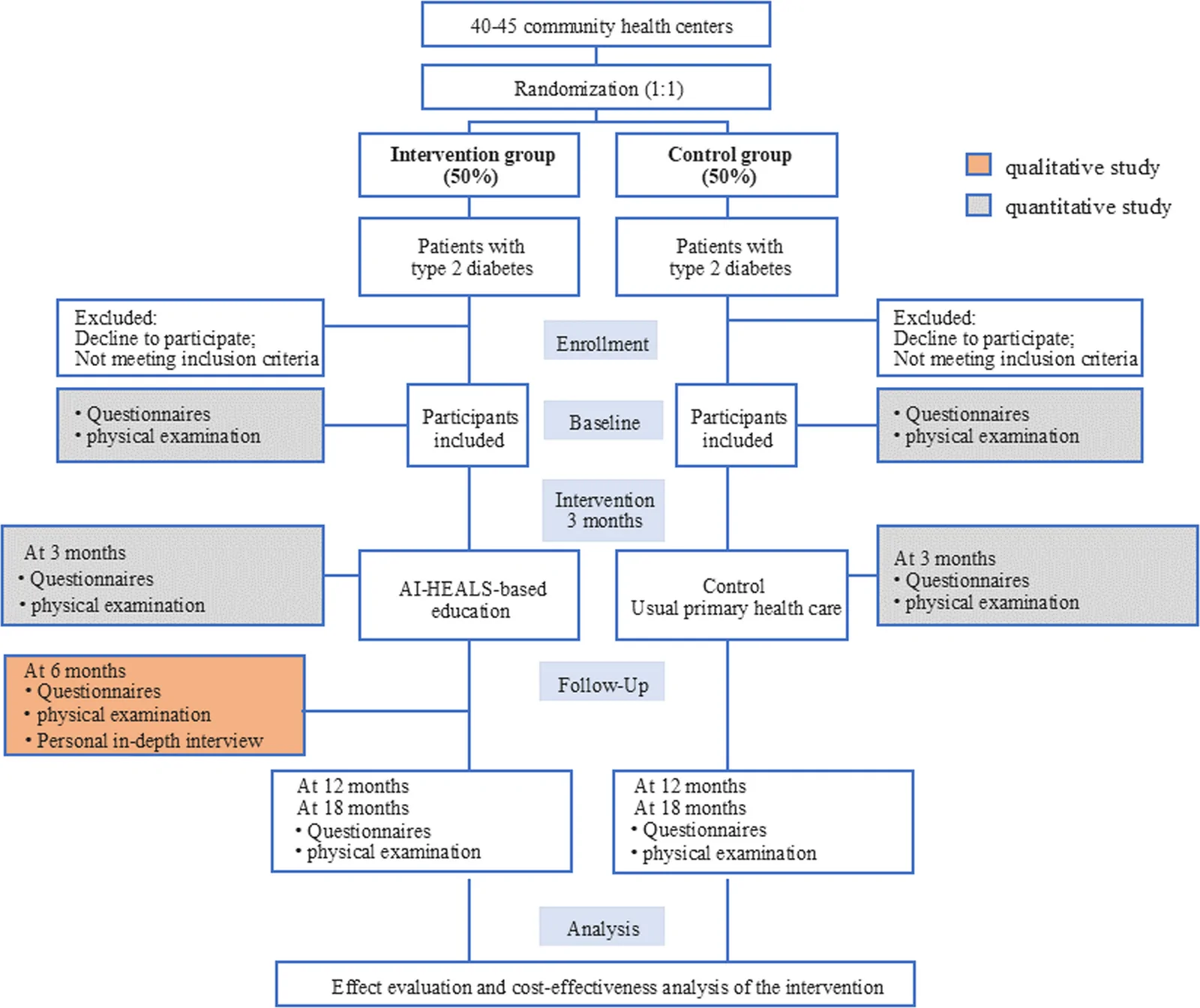
Flow chart of patient recruitment and study implementation

**Fig. 2:**
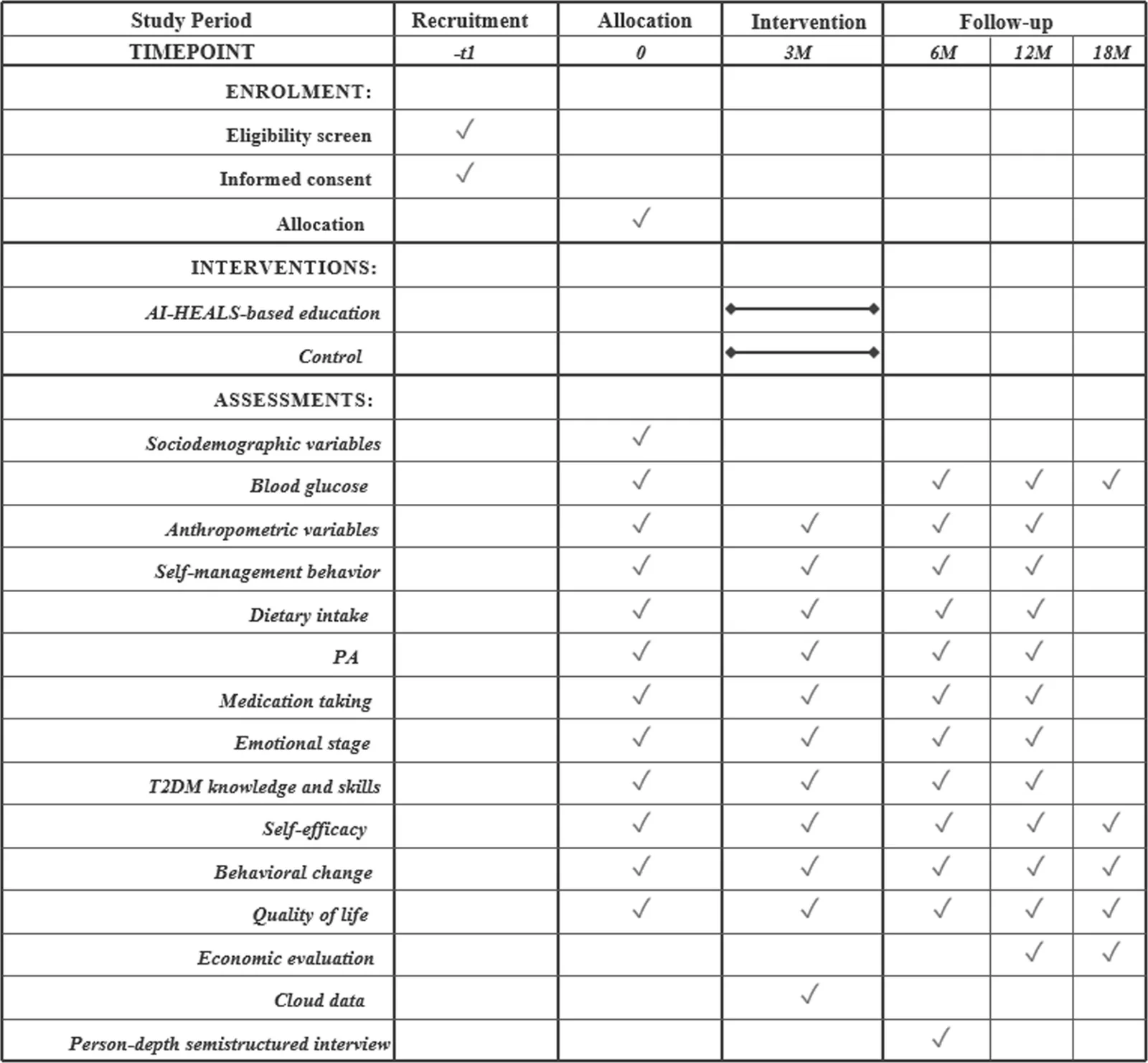
The schedule of enrolment, interventions, and assessments

This study will use a nested mixed-method study with a quantitative survey and a qualitative interview to gain a better understanding of the effect and acceptability of the AI and mHealth-based intervention program for T2DM. The research will take place from August 2023 to June 2025. The quantitative survey will be conducted as a community-based cluster randomized controlled trial with two parallel groups to compare the effectiveness of the AI-HEALS-based education program on T2DM control and self-management behaviors (such as medication use, dietary and physical activity modification). The intervention will last three months. Questionnaires and physical examinations will be conducted at baseline and at 3, 6, 12, and 18 months to examine changes in self management behaviors and blood glucose control. Personal in-depth interviews (the qualitative study) will be conducted at 6 months to explore participants’ experiences and feedback regarding the intervention. Cost-effectiveness analysis will be conducted at 12 and 18 months. The flow chart for the study shown in Figs. 1 and 2.

### Study setting and randomization

The study will be conducted in 40-45 community health centers (CHCs) in Beijing, China, as the unit of randomization. The community health center contains 8-10 people with T2DM. A computer-generated list of random numbers will assign clusters (CHCs) in a 1:1 ratio to an intervention group and a control group. All subjects in each cluster will receive the same intervention (SDPC plus AI-HEALS) or control (SDPC). Blinding of the participants or researchers cannot be guaranteed considering the nature of the intervention. The proportion of sociodemographic characteristics (such as gender, age, and location) between two groups will be evaluated at baseline to verify that the data are balanced and comparable. Study designer will generate the allocation sequence, CHCs staff will enroll participants, and study designer will assign participants to interventions.

### Population

The study population for our program is residents aged 18–75 years with T2DM in CHCs. The inclusion criteria are as follows: meet T2DM diagnostic criteria [65] (fasting plasma glucose ≥ 7.0 mmol/L or 2 h plasma glucose ≥ 11.1 mmol/L or HbA1c ≥ 6.5%); permanent residence in Beijing; ability to use smartphones and the WeChat chatting app; have not taken any psychotropic drugs prior to enrolment; have not participated in other studies; agree and able to adhere to the study. A history of type 1 diabetes, gestational diabetes, or secondary diabetes; severe diabetes complications; having radiotherapy or chemotherapy within the last six months; severe intellectual disability, Alzheimer's disease, or other mental disorders are key exclusion criteria. We have not yet started recruiting study participants, and we expect to begin recruitment in July 2023. After completing the entire questionnaire, participants will receive a gift of about 20 CNY. Participants will be informed that their participation is voluntary and that they can always opt out and switch to the SDPC.

### Effect size

The sample size calculation was based on assumptions prespecified in the original approved protocol, including a mean HbA1c of 7.13%, a standard deviation of 1.60%, an expected reduction in HbA1c to 6.50%, a two-sided significance level of 0.05, 90% power, and an intracluster correlation coefficient of 0.002. Based on the prespecified sample size calculation for a cluster-randomized trial [66], the required sample size was approximately 157 participants per group before allowing for loss to follow-up. After allowing for potential loss to follow-up, the planned sample size was increased to 200 participants per group, yielding a total target sample size of 400 participants.

### Intervention

#### Control arm: SDPC for patients with T2DM

Participants in the control group will receive standard health care from community doctors, including medical counselling, regular follow-up, assessment, health education, referral advice and medical examination.

#### Intervention arm: SDPC plus AI-HEALS-based T2DM online health education program

Participants will receive SDPC plus AI-HEALS-based intervention through the WeChat service platform "PKU Diabetes Butler" on their mobile phones, which consists of four components:

- 1. KBQA for T2DM knowledge. Participants will be encouraged to interact with a FAQRobot (Frequently Asked Questions Robot) to accurately and efficiently gain detailed knowledge about T2DM diet (recommended or not recommended foods, food nutrients, calories, glycemic index, and glycemic load), exercise (recommended or not recommended exercise, the metabolic equivalent of physical activities), and medication management (types of medicines and how to take them), removing time and distance limitations to health care consultations and increasing health care accessibility. In addition, the FAQRobot has an entertainment function, which can tell jokes and play games with patients to calm them down. After reviewing guidelines, professional literature, and consulting experts, we identified the source material and knowledge for the T2DM self-management KQBA. Subsequently, we uploaded the underlying data to the Bot Factory^22^ Dialogue AI Platform produced by Emotibot [67], which formed an automated Q&A bot with semantic understanding and automatic knowledge mining. The Q&A bot was linked to the WeChat platform "PKU Diabetes Butler". The backend stores the questions that participants asked as well as their usage behaviors (such as time and frequency) so that we can improve the knowledge graph regularly and assess the usage patterns of participants.
- 2. Recording and monitoring of physiological indicators as well as lifestyle behaviors. Participants will be encouraged to record information on physiological indicators (blood glucose, blood pressure), diet, physical activity, and medication-taking regularly on the platform, which stores and uploads the records electronically and enables long-term monitoring of blood glucose control and changes in self-management behaviors.
- 3. A reminder service for taking medications and monitoring blood glucose levels. Participants will be encouraged to enter the time they want to be reminded in the system, and the platform can remind them to take their medication or monitor blood glucose levels at specified time.
- 4. Automated and personalized messaging about diabetes self-management. Participants will receive 1–3 automated messages about T2DM control and management per week, the content of which will be based on multi-theory model (MTM) of health behavior change, and will contain knowledge of T2DM diet, exercise, and medication taking to increase health literacy and to encourage behavioral change. Furthermore, based on the questions users ask the FAQRobot, the system will accurately push additional, tailored articles. Researchers will also regularly log into the backend to check the number, type and time of articles read by the participants in order to tailor the content and frequency of messages to their reading patterns.

All user data will be kept confidential. Participants have the option to withdraw from the study at any time. Adverse events related to the intervention will be recorded and reported in accordance with local procedures.

### Outcomes

Primary outcome: Change of HbA1c from baseline to 3, 6, 12, and 18-month follow-up.

Secondary outcomes:

- 1. Modifications of self-management behaviors: dietary, physical activity, blood glucose monitoring, and medication adherence.
- 2. Social cognition and psychology: diabetes health literacy, self-efficacy, depression, anxiety and stress.
- 3. Metabolic control: self-reported fasting plasma glucose or 2 h plasma glucose, dose of insulin and medicine, other indicators such as blood pressure, blood lipids, and BMI.
- 4. Economic evaluation: A comparison of incremental cost-effectiveness ratios (ICERs) will be conducted between the AI-HEALS-based intervention and usual care of T2DM. Therefore, health-related quality of life (HRQoL), healthcare utilization, including medical consultations, use of healthcare (outpatient, hospitalized, and primary care services), medicine purchases, accident visits, and direct non-medical costs (diabetes-related transportation, lodging) will be collected.

Additionally, the rate of recruitment and drop-out, as well as the reasons for drop-out, will be gathered. To determine whether the data is homogeneous, demographic characteristics and study outcomes will be compared between people dropping out and participants resistant to the entire trial during data analysis.

### Variables measurement

Sociodemographic variables will be obtained at base- line. Physical examinations and questionnaire surveys will be completed at baseline, 3, 6, 12, and 18 months (Table 1).

**Table 1:** Data collection components and collection timeline

**Type of research**	**Data collection component**			**Timepoint**				
				**0 M**	**3 M**	**6 M**	**12 M**	**18 M**
Quantitative research	Sociodemographic variables	sex, age, marital status, location, family average household, educational level, occupation, clinical information		√				
	Blood glucose	HbA1c levels		√	√	√	√	√
	Anthropometric variables	height, weight, waist circumference, and blood pressure		√	√	√	√	√
	Self-management behavior	Questionnaire: SDSCA		√	√	√	√	√
	Dietary intake	Questionnaire: self-administered diabetes eating behavior questionnaire		√	√	√	√	√
	PA	Questionnaire: IPAQ-SF		√	√	√	√	√
	Medication taking	Questionnaire: ARMS		√	√	√	√	√
	Emotional stage	Questionnaire: GAD-7, PHQ9, and PSS-4		√	√	√	√	√
	T2DM knowledge and skills	Questionnaire: HLS-SF12 and self-designed 9-item diabetes self-management skills scale		√	√	√	√	√
	Self-efficacy	Questionnaire: self-designed diabetes self-management scale		√	√	√	√	√
	Behavioral change	Questionnaire: self-designed 31-item MTM scale for health behavior change		√	√	√	√	√
	Quality of life	Questionnaire: EQ-5D-5L		√	√	√	√	√
	Economic evaluation	healthcare utilization (medical consultations, use of out- patient, hospitalized, and primary care services, medicine expenditures, accident visits, and lost productivity)					√	√
	Cloud data	KQBA interaction records and user reading behavior data		√	√			
Qualitative research	Person-in-depth semistruc- tured interview	User experience of the AI-HEALS				√		

Sociodemographic variables: sex, age, marital status, location, family average household, educational level, and occupation will be gathered, as well as clinical information such as the duration of T2DM, detailed medical history, and current diabetes medications.

Measurement of blood glucose: HbA1c levels will be measured by drawing venous blood fasting.

Anthropometric variables: height, weight, waist circumference, and blood pressure of each participant will be measured twice by certified instruments. Height and body weight will be measured using an adequately calibrated certified electronic instrument. Divide weight (kg) by height (m) to calculate the BMI. Waist circumference will be measured at the level of the umbilicus using a tape measure. As regards blood pressure, systolic and diastolic BP will be measured by a validated automated sphygmomanometer.

#### Questionnaires

Self-management behavior will be evaluated by the Summary of Diabetes Self Care Activities (SDSCA). SDSCA is an 11-item brief self-report questionnaire for measuring levels of T2DM self-management behaviors with diet (5 items), exercise (2 items), blood sugar testing (2 items), and foot care( 2 items) in last 7 days, which has great reliability and validity for both research and practice [68].

Dietary intake will be assessed by self-administered diabetes eating behavior questionnaire, including sugar and fat (5 items), fruit and vegetable (5 items), oil and salt eating habits (4 items), cooking and dining habits (4 items), and dietary monitoring behavior (4 items).

Physical activity during the last 7 days will be assessed by International Physical Activity Questionnaire Short Form (IPAQ-SF), including items for time spent on slight, moderate, vigorous activities or sedentary behavior [69].

Medication taking behavior will be evaluated by the Adherence to Refills and Medications Scale (ARMS) [70], a 12-item scale containing taking medications as prescribed and refilling medications on schedule, which has great validity and reliability for measuring medication adherence.

Emotional state will be measured by the 7-item Generalized Anxiety Disorder (GAD-7) [71], the Patient Health Questionnaire 9 (PHQ9) [72], and the 4-item Perceived Stress Scale (PSS-4) [73], which reflect participants’ levels of anxiety, depression, and stress over the last two weeks.

Health literacy will be evaluated by 12-item short-form health literacy scale (HLS-SF12) [74], which includes 12 dimensions of health literacy with 12 questions about finding, understanding, judging, and applying health information about disease management, prevention, and health promotion.

Diabetes self-management skills will be assessed by a self-designed 9-item scale for people with T2DM, including determining if their weight is normal, calculating their daily calorie needs, designing personalized recipes, determining the size of the effect of regular food on blood sugar, adjusting their diet according to the amount of exercise they do, developing their own exercise plan, judging the intensity of various exercises, and knowing how to avoid and deal with hypoglycemia.

Self-efficacy will be measured by self-designed diabetes self-management scale, which reflects the confidence to do diabetes self-management behaviors, including diet (5 items), physical activity (7 items), identify and manage hypoglycemia (2 items) and access medical services (2 items).

Change of self-management behaviors will be evaluated by a self-designed 31-item MTM scale for health behavior change, including participatory dialogue, behavioral confidence, changes in the physical environment and social environment, emotional change, and behavioral change.

Quality of life will be measured by five-level EuroQol five-dimensional questionnaire (EQ-5D-5L) [75], which comprises five dimensions about mobility, self-care, usual activities, pain/discomfort, and anxiety/depression.

Cloud data: The cloud data from the AI-HEALS will be used to evaluate users' FAQRobot interaction records and reading behavior data on a weekly basis. The FAQRobot interaction records include user ID, user questions, bot answers, human–computer interaction time (accurate to the second) and outgoing talk module (knowledge reasoning engine, and common standard questions). User reading behavior data includes article clicks and reads, reading time per article, average effective reading rate (patients/articles), follow rate and unfollow rate of the WeChat service platform "PKU Diabetes Butler", and article Retweets. A collaboration agreement for this project has been made with the Emotibot company. The data will not include users' personal information, and will be used exclusively for this project and cannot be used for other purposes without authorization.

### Economic evaluation

To assess the efficacy of this mHealth education program on health equality, the economic evaluation will be reported as ICERs and compared between the AI-HEALS-based intervention and traditional T2DM primary care. During the trial, the economic cost of healthcare utilization (medical consultations, use of outpatient, hospitalized, and primary care services, medicine expenditures, accident visits, and lost productivity) will be assessed per case, as well as the cost of project implementation (AI-HEALS-based interventions (plus primary usual care activities) versus primary usual care activities). Utility will be evaluated in terms of HRQoL, with data from the EQ-5D-5L used to calculate health utility values.

### Qualitative analysis

Semistructured interviews of participants will be conducted 6 months after baseline to investigate feedback, including program appeal, acceptability, usability, and participant satisfaction. The interview data will provide us a better understanding of the factors that drive engagement and behavioral changes in participants. We will invite participants to a 40-min telephone-based or face-to-face personal in-depth interview. Participants will be selected considering the sociodemographic variables including sex, age, location, educational level and the duration of T2DM. Topics about personal experience, recommendations for the developments of the AI-HEALS as well as motivating factors and difficulties of maintaining self-management behaviors are all encouraged to be discussed. Participants will be recruited successively until thematic saturation has been reached. Interview audio files will be transcribed verbatim and de-identified, and analysed by NVivo (version 12, QRS International, Doncaster, Australia) using the theoretical domains framework. The qualitative data and quantitative data will be incorporated to better explain the results at multiple stages during the trial.

### Statistical analysis

Data from the quantitative study will be recorded in the designed questionnaire and double-parallelly entered into Epidata 3.1 (Version 15.0.5, Odense, Denmark). Variables analysis will be based on the premise of intention to treat. Continuous variables will be presented as mean ± SD (M(P_25_, P_75_), if they are abnormal distribution), and be compared using Student's t test or one-way ANOVA (or non-parametric tests if necessary). Data distribution will be identified by a Kolmogorov–Smirnov test. Categorical data reported as n(percentage of sample) will be compared using χ2 tests. With regard to the cross-sectional analysis, linear regression, logistic regression and structural equation model (SEM) will be conducted to explore the influence factors of behaviors pattern and mHealth usage pattern evaluated by the group-based trajectory model (GBTM). For longitudinal data, a generalized linear mixed model (GLMM) will be used to identify the effectiveness of the AI and mHealth-based intervention in behavioral changes and diabetic control. Set an alpha risk of 0.05 for two-sided tests. Data will be analysed using IBM SPSS Statistics version 24.0 (SPSS Inc., an IBM Company, Chicago, Illinois, USA), Stata (version 14.0, Stata Corporation, College Station, TX) and M*plus* (version 7.4, Muthén & Muthén, Los Angeles, CA, USA).

### Study management

Prior to the study, the investigators will be trained and assessed comprehensively. Throughout the study, the study subjects will be registered and coded to minimize the occurrence of missing visits during the follow-up period. To maintain consistency between the intervention and control groups, survey time and questionnaire will be kept substantially identical. During data analysis, a double-entry checking procedure will be implemented to enhance the quality of data entry, and experts will be consulted to select appropriate statistical methods. If finding abnormal values, the original questionnaires will be checked and verified for accuracy to proceed to the analysis. Additionally, the qualitative data will be collated and proofread by two researchers to ensure reliability in the analysis of the qualitative findings.

The research leaders and primary investigators will supervise research implementers regularly to ensure the correct implementation of all research procedures and to guarantee the accuracy of the collected data. If investigators are failure to adhere to predefined survey and follow-up standards, research leaders will terminate the trial, revise the randomization scheme and recollect data after the modification is completed. In the event of fundamental or widespread design errors or inaccuracies in the research questionnaire during the survey process, the researchers will halt the trial to modify and redesign the questionnaire. After the necessary revisions were completed, the revised questionnaire will be then redistributed to participants.

The data monitoring committee (DMC) will be composed of at least two members from the Biomedical Ethics Committee of Peking University, who have no conflicts of interest with our study. The DMC will conduct regular monitoring and review of the study, and will report the findings to the Ethics Committee. This process will operate independently of the primary investigators. If the DMC identifies deviations from the approved research protocol or unauthorized changes to the study procedures during the research process, they have the authority to suspend or terminate the study. Furthermore, during the research, the project researchers are responsible for promptly reporting any serious adverse events (SAE) or adverse events (AE) to the Ethics Committee. If any participants experience AE or SAE, regardless of their relationship to the research intervention or whether the intervention has been implemented, the researchers must notify the investigators within 24 h of the event and terminate the trial. Additionally, in the event of serious program bugs or significant deficiencies in user interaction during the participant's engagement with AI, the researchers must interrupt the trial, redesign the study, and rectify the issues with the AI program.

## Discussion

As the Internet and smartphones have advanced, an increasing number of people are turning to mobile Internet to obtain information, and mHealth has been shown to be a cost-effective way to promote self-management behaviors in T2DM primary care. AI and mHealth-based programs have shown great promise in diabetes management, such as predicting blood glucose levels, monitoring medication dosages, identifying high-risk patients, and assisting in diabetes diagnosis. KBQA and AI-based reminding and monitoring programs in CHCs can answer patients’ questions and provide real-time feedback on their blood glucose levels, dietary patterns, medication taking and exercise, removing time and distance barriers to health care consultations and increase health care accessibility. AI can also interact with patients in a playful manner, improving their mood and adherence to treatment. An AI-based diabetes self-management assistant would be an excellent tool for both clinicians and patients as the GPT model is promoted and developed.

Since there have been few studies on integrating AI technology, especially intelligent question and answer systems, into T2DM community interventions. The findings of this study will indicate the feasibility and effecacy of the AI-based KBQA, monitoring, recording and alerting system for individualized T2DM interventions. The findings will provide meaningful insights into refining AI-based T2DM management and improving the usability of diabetes mHealth software.

## Supplementary Information

**Additional file 1.**

## Data Availability

Research leaders and data analysts have access to the final data set. The datasets used and/or analysed during the current study are available from the corresponding author on reasonable request.

## Abbreviations

AI-HEALS: Artificial Intelligence-based Health Education Accurately Linking System
T2DM: Type 2 diabetes
AI: Artificial intelligence
KG: Knowledge graph
QA: Question and answering
SDPC: Standard diabetes primary care
KBQA: Knowledge-based question answering
PA: Physical activity
SMBG: Self-monitoring of blood glucose
mHealth: Mobile health
DMO: Digital medicine offering
ML: Machine learning
NLP: Natural language processing
FL: Fuzzy logic; ES: expert systems
KB: Knowledge base
ADA: American Diabetes Association
CHCs: Community health centers
ICC: Intraclass correlation
SD: Standard deviation
FAQRobot: Frequently Asked Questions Robot
MTM: Multi-theory model
ICERs: Incremental cost-effectiveness ratios
HRQoL: Health-related quality of life
SDSCA: Summary of Diabetes Self Care Activities
IPAQ-SF: International Physical Activity Questionnaire Short Form
ARMS: The Adherence to Refills and Medications Scale
GAD-7: 7-Item Generalized Anxiety Disorder
PHQ9: The Patient Health Questionnaire 9
PSS-4: The 4-item Perceived Stress Scale
HLS-SF12: 12-Item short-form health literacy scale
EQ-5D-5L: Five-level EuroQol five-dimensional questionnaire
SEM: Structural equation model
GBTM: Group-based trajectory model
GLMM: Generalized linear mixed model
DMC: Data monitoring committee
SAE: Serious adverse events
AE: Adverse events

## References

[1] International Diabetes Federation. IDF Diabetes Atlas teB, Belgium: International Diabetes Federation, 2021.

[2] Buse JB, Wexler DJ, Tsapas A, Rossing P, Mingrone G, Mathieu C, D’Alessio DA, Davies MJ. 2019 Update to: management of hyperglycemia in Type 2 Diabetes, 2018. a Consensus Report by the American Diabetes Association (ADA) and the European Association for the Study of Diabetes (EASD). Diabetes Care. 2020;43(2):487–93. 10.2337/dci19-0066 PMC6971782 31857443

[3] Toi PL, Anothaisintawee T, Chaikledkaew U, Briones JR, Reutrakul S, Thakkinstian A. Preventive role of diet interventions and dietary factors in Type 2 diabetes mellitus: an umbrella review. Nutrients. 2020;12(9):2722. 10.3390/nu12092722 PMC7551929 32899917

[4] Papamichou D, Panagiotakos DB, Itsiopoulos C. Dietary patterns and management of type 2 diabetes: a systematic review of randomised clinical trials. Nutr Metab Cardiovasc Dis. 2019;29(6):531–43. 10.1016/j.numecd.2019.02.004 30952576

[5] Hemmingsen B, Gimenez-Perez G, Mauricio D, Roque IFM, Metzendorf MI, Richter B. Diet, physical activity or both for prevention or delay of type 2 diabetes mellitus and its associated complications in people at increased risk of developing type 2 diabetes mellitus. Cochrane Database Syst Rev. 2017;12:CD003054. 10.1002/14651858.CD003054.pub4 PMC6486271 29205264

[6] Russell WR, Baka A, Bjorck I, Delzenne N, Gao D, Griffiths HR, Hadjilucas E, Juvonen K, Lahtinen S, Lansink M, et al. Impact of diet composition on blood glucose regulation. Crit Rev Food Sci Nutr. 2016;56(4):541–90. 10.1080/10408398.2013.792772 24219323

[7] Esposito K, Maiorino MI, Ciotola M, Di Palo C, Scognamiglio P, Gicchino M, Petrizzo M, Saccomanno F, Beneduce F, Ceriello A, et al. Effects of a Mediterranean-style diet on the need for antihyperglycemic drug therapy in patients with newly diagnosed type 2 diabetes: a randomized trial. Ann Intern Med. 2009;151(5):306–14. 10.7326/0003-4819-151-5-200909010-00004 19721018

[8] Esposito K, Maiorino MI, Petrizzo M, Bellastella G, Giugliano D. The effects of a Mediterranean diet on the need for diabetes drugs and remission of newly diagnosed type 2 diabetes: follow-up of a randomized trial. Diabetes Care. 2014;37(7):1824–30. 10.2337/dc13-2899 24722497

[9] Jannasch F, Kroger J, Schulze MB. dietary patterns and Type 2 Diabetes: a systematic literature review and meta-analysis of prospective studies. J Nutr. 2017;147(6):1174–82. 10.3945/jn.116.242552 28424256

[10] Schwingshackl L, Hoffmann G. Diet quality as assessed by the healthy eating index, the alternate healthy eating index, the dietary approaches to stop hypertension score, and health outcomes: a systematic review and meta-analysis of cohort studies. J Acad Nutr Diet. 2015;115(5):780-800 e785. 10.1016/j.jand.2014.12.009 25680825

[11] Esposito K, Chiodini P, Maiorino MI, Bellastella G, Panagiotakos D, Giugliano D. Which diet for prevention of type 2 diabetes? a meta-analysis of prospective studies. Endocrine. 2014;47(1):107–16. 10.1007/s12020-014-0264-4 24744219

[12] Lean ME, Leslie WS, Barnes AC, Brosnahan N, Thom G, McCombie L, Peters C, Zhyzhneuskaya S, Al-Mrabeh A, Hollingsworth KG, et al. Primary care-led weight management for remission of type 2 diabetes (DiRECT): an open-label, cluster-randomised trial. Lancet. 2018;391(10120):541–51. 10.1016/S0140-6736(17)33102-1 29221645

[13] Lim EL, Hollingsworth KG, Aribisala BS, Chen MJ, Mathers JC, Taylor R. Reversal of type 2 diabetes: normalisation of beta cell function in association with decreased pancreas and liver triacylglycerol. Diabetologia. 2011;54(10):2506–14. 10.1007/s00125-011-2204-7 PMC3168743 21656330

[14] Gregg EW, Chen H, Wagenknecht LE, Clark JM, Delahanty LM, Bantle J, Pownall HJ, Johnson KC, Safford MM, Kitabchi AE, et al. Association of an intensive lifestyle intervention with remission of type 2 diabetes. JAMA. 2012;308(23):2489–96. 10.1001/jama.2012.67929 PMC4771522 23288372

[15] Elhayany A, Lustman A, Abel R, Attal-Singer J, Vinker S. A low carbohydrate Mediterranean diet improves cardiovascular risk factors and diabetes control among overweight patients with type 2 diabetes mellitus: a 1-year prospective randomized intervention study. Diabetes Obes Metab. 2010;12(3):204–9. 10.1111/j.1463-1326.2009.01151.x 20151996

[16] Rock CL, Flatt SW, Pakiz B, Taylor KS, Leone AF, Brelje K, Heath DD, Quintana EL, Sherwood NE. Weight loss, glycemic control, and cardiovascular disease risk factors in response to differential diet composition in a weight loss program in type 2 diabetes: a randomized controlled trial. Diabetes Care. 2014;37(6):1573–80. 10.2337/dc13-2900 PMC4392939 24760261

[17] Wheeler ML, Dunbar SA, Jaacks LM, Karmally W, Mayer-Davis EJ, Wylie-Rosett J, Yancy WS Jr. Macronutrients, food groups, and eating patterns in the management of diabetes: a systematic review of the literature, 2010. Diabetes Care. 2012;35(2):434–45. 10.2337/dc11-2216 PMC3263899 22275443

[18] Barnard ND, Cohen J, Jenkins DJ, Turner-McGrievy G, Gloede L, Green A, Ferdowsian H. A low-fat vegan diet and a conventional diabetes diet in the treatment of type 2 diabetes: a randomized, controlled, 74-wk clinical trial. Am J Clin Nutr. 2009;89(5):1588S-1596S. 10.3945/ajcn.2009.26736H PMC2677007 19339401

[19] Viguiliouk E, Kendall CW, Kahleova H, Rahelic D, Salas-Salvado J, Choo VL, Mejia SB, Stewart SE, Leiter LA, Jenkins DJ, et al. Effect of vegetarian dietary patterns on cardiometabolic risk factors in diabetes: a systematic review and meta-analysis of randomized controlled trials. Clin Nutr. 2019;38(3):1133–45. 10.1016/j.clnu.2018.05.032 29960809

[20] Soare A, Del Toro R, Khazrai YM, Di Mauro A, Fallucca S, Angeletti S, Skrami E, Gesuita R, Tuccinardi D, Manfrini S, et al. A 6-month follow-up study of the randomized controlled Ma-Pi macrobiotic dietary intervention (MADIAB trial) in type 2 diabetes. Nutr Diabetes. 2016;6(8): e222. 10.1038/nutd.2016.29 PMC5022142 27525817

[21] Chiavaroli L, Lee D, Ahmed A, Cheung A, Khan TA, Blanco S, Mejia, Mirrahimi A, Jenkins DJA, Livesey G et al: Effect of low glycaemic index or load dietary patterns on glycaemic control and cardiometabolic risk factors in diabetes: systematic review and meta-analysis of randomised controlled trials. BMJ 2021, 374:n1651. 10.1136/bmj.n1651 PMC8336013 34348965

[22] Livesey G, Taylor R, Livesey HF, Buyken AE, Jenkins DJA, Augustin LSA, Sievenpiper JL, Barclay AW, Liu S, Wolever TMS, et al. Dietary glycemic index and load and the risk of Type 2 diabetes: assessment of causal relations. Nutrients. 2019;11(6):1436. 10.3390/nu11061436 PMC6628270 31242690

[23] Livesey G, Taylor R, Livesey HF, Buyken AE, Jenkins DJA, Augustin LSA, Sievenpiper JL, Barclay AW, Liu S, Wolever TMS, et al. Dietary glycemic index and load and the risk of Type 2 diabetes: a systematic review and updated meta-analyses of prospective cohort studies. Nutrients. 2019;11(6):1280. 10.3390/nu11061280 PMC6627334 31195724

[24] Ojo O, Ojo OO, Adebowale F, Wang XH. The effect of dietary glycaemic index on glycaemia in patients with Type 2 diabetes: a systematic review and meta-analysis of randomized controlled trials. Nutrients. 2018;10(3):373. 10.3390/nu10030373 PMC5872791 29562676

[25] Boussageon R, Roustit M, Gueyffier F, Tudrej BV, Rehman MB. Type 2 diabetes. Lancet. 2018;391(10127):1261. 10.1016/S0140-6736(18)30702-5 29619958

[26] Collaboration NCDRF: Worldwide trends in body-mass index, underweight, overweight, and obesity from 1975 to 2016: a pooled analysis of 2416 population-based measurement studies in 128.9 million children, adolescents, and adults. Lancet 2017, 390(10113):2627–2642. 10.1016/S0140-6736(17)32129-3 PMC5735219 29029897

[27] Chatterjee S, Khunti K, Davies MJ. Type 2 diabetes. Lancet. 2017;389(10085):2239–51. 10.1016/S0140-6736(17)30058-2 28190580

[28] Smith AD, Crippa A, Woodcock J, Brage S. Physical activity and incident type 2 diabetes mellitus: a systematic review and dose-response meta-analysis of prospective cohort studies. Diabetologia. 2016;59(12):2527–45. 10.1007/s00125-016-4079-0 PMC6207340 27747395

[29] Umpierre D, Ribeiro PA, Kramer CK, Leitao CB, Zucatti AT, Azevedo MJ, Gross JL, Ribeiro JP, Schaan BD. Physical activity advice only or structured exercise training and association with HbA1c levels in type 2 diabetes: a systematic review and meta-analysis. JAMA. 2011;305(17):1790–9. 10.1001/jama.2011.576 21540423

[30] Amanat S, Ghahri S, Dianatinasab A, Fararouei M, Dianatinasab M. Exercise and Type 2 Diabetes. Adv Exp Med Biol. 2020;1228:91–105. 10.1007/978-981-15-1792-1_6 32342452

[31] Balducci S, Sacchetti M, Haxhi J, Orlando G, D’Errico V, Fallucca S, Menini S, Pugliese G. Physical exercise as therapy for type 2 diabetes mellitus. Diabetes Metab Res Rev. 2014;30(Suppl 1):13–23. 10.1002/dmrr.2514 24353273

[32] Kanaley JA, Colberg SR, Corcoran MH, Malin SK, Rodriguez NR, Crespo CJ, Kirwan JP, Zierath JR. Exercise/physical activity in individuals with Type 2 Diabetes: a consensus statement from the American college of sports medicine. Med Sci Sports Exerc. 2022;54(2):353–68. 10.1249/MSS.0000000000002800 PMC8802999 35029593

[33] Davies MJ, D’Alessio DA, Fradkin J, Kernan WN, Mathieu C, Mingrone G, Rossing P, Tsapas A, Wexler DJ, Buse JB. Management of Hyperglycemia in Type 2 Diabetes, 2018. a consensus report by the American Diabetes Association (ADA) and the European Association for the Study of Diabetes (EASD). Diabetes Care. 2018;41(12):2669–701. 10.2337/dci18-0033 PMC6245208 30291106

[34] Clark JE. Diet, exercise or diet with exercise: comparing the effectiveness of treatment options for weight-loss and changes in fitness for adults (18–65 years old) who are overfat, or obese; systematic review and meta-analysis. J Diabetes Metab Disord. 2015;14:31. 10.1186/s40200-015-0154-1 PMC4429709 25973403

[35] Alonso-Dominguez R, Gomez-Marcos MA, Patino-Alonso MC, Sanchez-Aguadero N, Agudo-Conde C, Castano-Sanchez C, Garcia-Ortiz L, Recio-Rodriguez JI. Effectiveness of a multifactorial intervention based on an application for smartphones, heart-healthy walks and a nutritional workshop in patients with type 2 diabetes mellitus in primary care (EMID): study protocol for a randomised controlled trial. BMJ Open. 2017;7(9): e016191. 10.1136/bmjopen-2017-016191 PMC5640135 28912193

[36] Siegel KR, Ali MK, Zhou X, Ng BP, Jawanda S, Proia K, Zhang X, Gregg EW, Albright AL, Zhang P. Cost-effectiveness of interventions to manage diabetes: has the evidence changed since 2008? Diabetes Care. 2020;43(7):1557–92. 10.2337/dci20-0017 33534729

[37] Jiang X, Ming WK, You JH. The cost-effectiveness of digital health interventions on the management of cardiovascular diseases: systematic review. J Med Internet Res. 2019;21(6): e13166. 10.2196/13166 PMC6601257 31210136

[38] de Batlle J, Massip M, Vargiu E, Nadal N, Fuentes A, Ortega Bravo M, Miralles F, Barbe F, Torres G. Group CO-L: implementing mobile health-enabled integrated care for complex chronic patients: intervention effectiveness and cost-effectiveness study. JMIR Mhealth Uhealth. 2021;9(1): e22135. 10.2196/22135 PMC7843204 33443486

[39] Wu X, Guo X, Zhang Z. The efficacy of mobile phone apps for lifestyle modification in diabetes: systematic review and meta-analysis. JMIR Mhealth Uhealth. 2019;7(1): e12297. 10.2196/12297 PMC6350094 30664494

[40] Lunde P, Nilsson BB, Bergland A, Kvaerner KJ, Bye A. The effectiveness of smartphone apps for lifestyle improvement in noncommunicable diseases: systematic review and meta-analyses. J Med Internet Res. 2018;20(5): e162. 10.2196/jmir.9751 PMC5960039 29728346

[41] Holmen H, Torbjornsen A, Wahl AK, Jenum AK, Smastuen MC, Arsand E, Ribu L. A mobile health intervention for self-management and lifestyle change for persons with Type 2 Diabetes, part 2: one-year results from the norwegian randomized controlled trial renewing health. JMIR Mhealth Uhealth. 2014;2(4): e57. 10.2196/mhealth.3882 PMC4275495 25499872

[42] Poppe L, De Bourdeaudhuij I, Verloigne M, Shadid S, Van Cauwenberg J, Compernolle S, Crombez G. Efficacy of a self-regulation-based electronic and mobile health intervention targeting an active lifestyle in adults having Type 2 Diabetes and in adults aged 50 years or older: two randomized controlled trials. J Med Internet Res. 2019;21(8): e13363. 10.2196/13363 PMC6696857 31376274

[43] Frias J, Virdi N, Raja P, Kim Y, Savage G, Osterberg L. Effectiveness of digital medicines to improve clinical outcomes in patients with uncontrolled hypertension and Type 2 Diabetes: prospective, open-label, cluster-randomized pilot clinical trial. J Med Internet Res. 2017;19(7): e246. 10.2196/jmir.7833 PMC5527253 28698169

[44] Hansel B, Giral P, Gambotti L, Lafourcade A, Peres G, Filipecki C, Kadouch D, Hartemann A, Oppert JM, Bruckert E, et al. A Fully automated web-based program improves lifestyle habits and HbA1c in patients with Type 2 Diabetes and abdominal obesity: randomized trial of patient e-coaching nutritional support (The ANODE Study). J Med Internet Res. 2017;19(11): e360. 10.2196/jmir.7947 PMC5700402 29117929

[45] Eberle C, Lohnert M, Stichling S. Effectiveness of disease-specific mhealth apps in patients with diabetes mellitus: scoping review. JMIR Mhealth Uhealth. 2021;9(2): e23477. 10.2196/23477 PMC7920757 33587045

[46] Abhari S, Niakan Kalhori SR, Ebrahimi M, Hasannejadasl H, Garavand A. Artificial Intelligence applications in Type 2 Diabetes mellitus care: focus on machine learning methods. Healthc Inform Res. 2019;25(4):248–61. 10.4258/hir.2019.25.4.248 PMC6859270 31777668

[47] Kagawa R, Kawazoe Y, Ida Y, Shinohara E, Tanaka K, Imai T, Ohe K. Development of Type 2 Diabetes Mellitus phenotyping framework using expert knowledge and machine learning approach. J Diabetes Sci Technol. 2017;11(4):791–9. 10.1177/1932296816681584 PMC5588819 27932531

[48] Zheng T, Xie W, Xu L, He X, Zhang Y, You M, Yang G, Chen Y. A machine learning-based framework to identify type 2 diabetes through electronic health records. Int J Med Inform. 2017;97:120–7. 10.1016/j.ijmedinf.2016.09.014 PMC5144921 27919371

[49] Anderson AE, Kerr WT, Thames A, Li T, Xiao J, Cohen MS. Electronic health record phenotyping improves detection and screening of type 2 diabetes in the general United States population: a cross-sectional, unselected, retrospective study. J Biomed Inform. 2016;60:162–8. 10.1016/j.jbi.2015.12.006 PMC5679210 26707455

[50] Sudharsan B, Peeples M, Shomali M. Hypoglycemia prediction using machine learning models for patients with type 2 diabetes. J Diabetes Sci Technol. 2015;9(1):86–90. 10.1177/1932296814554260 PMC4495530 25316712

[51] Cai J, Li C, Liu Z, Du J, Ye J, Gu Q, Xu J. Predicting DPP-IV inhibitors with machine learning approaches. J Comput Aided Mol Des. 2017;31(4):393–402. 10.1007/s10822-017-0009-6 28155089

[52] Zhang YF, Tian Y, Zhou TS, Araki K, Li JS. Integrating HL7 RIM and ontology for unified knowledge and data representation in clinical decision support systems. Comput Methods Programs Biomed. 2016;123:94–108. 10.1016/j.cmpb.2015.09.020 26474836

[53] Luo S, Chen S, Pan L, Zhang T, Han L, Wang Y, Safi QG. Exploring the effects of intervention for those at high risk of developing type 2 diabetes using a computer simulation. Comput Biol Med. 2014;53:105–14. 10.1016/j.compbiomed.2014.05.015 25129022

[54] Luo G. Automatically explaining machine learning prediction results: a demonstration on type 2 diabetes risk prediction. Health Inf Sci Syst. 2016;4:2. 10.1186/s13755-016-0015-4 PMC4782293 26958341

[55] Lee BJ, Kim JY. Identification of Type 2 Diabetes risk factors using phenotypes consisting of anthropometry and triglycerides based on machine learning. IEEE J Biomed Health Inform. 2016;20(1):39–46. 10.1109/JBHI.2015.2396520 25675467

[56] Rau HH, Hsu CY, Lin YA, Atique S, Fuad A, Wei LM, Hsu MH. Development of a web-based liver cancer prediction model for type II diabetes patients by using an artificial neural network. Comput Methods Programs Biomed. 2016;125:58–65. 10.1016/j.cmpb.2015.11.009 26701199

[57] Rigla M, Garcia-Saez G, Pons B, Hernando ME. Artificial intelligence methodologies and their application to diabetes. J Diabetes Sci Technol. 2018;12(2):303–10. 10.1177/1932296817710475 PMC5851211 28539087

[58] Lan Y, He S, Liu K, Zeng X, Liu S, Zhao J. Path-based knowledge reasoning with textual semantic information for medical knowledge graph completion. BMC Med Inform Decis Mak. 2021;21(Suppl 9):335. 10.1186/s12911-021-01622-7 PMC8628388 34844576

[59] Chang D, Chen M, Liu C, Liu L: DiaKG: an Annotated Diabetes Dataset for Medical Knowledge Graph Construction. In China Conference on Knowledge Graph and Semantic Computing (pp 308–314) Springer, Singapore 2021.

[60] Huang X, Zhang J, Xu Z, Ou L, Tong J. A knowledge graph based question answering method for medical domain. PeerJ Comput Sci. 2021;7: e667. 10.7717/peerj-cs.667 PMC8444078 34604514

[61] Yin Y, Zhang L, Wang Y, Wang M, Zhang Q, Li GZ. Question answering system based on knowledge graph in traditional chinese medicine diagnosis and treatment of viral Hepatitis B. Biomed Res Int. 2022;2022:7139904. 10.1155/2022/7139904 PMC8860556 35198638

[62] Xiu X, Qian Q, Wu S. Construction of a digestive system tumor knowledge graph based on chinese electronic medical records: development and usability study. JMIR Med Inform. 2020;8(10): e18287. 10.2196/18287 PMC7578820 33026359

[63] Singh Rawat BP, Li F, Yu H. Clinical judgement study using question answering from electronic health records. Proc Mach Learn Res. 2019;106:216–29. PMC6939641 31897452

[64] Chen Y SA, Chen C H, et al. : Personalized food recommendation as constrained question answering over a large-scale food knowledge graph. Proceedings of the 14th ACM International Conference on Web Search and Data Mining 2021: 544–552.

[65] Society CD: Guideline for the prevention and treatment of type 2 diabetes mellitus in China (2020 edition). Chinese Journal of Diabetes Mellitus 2021, 13(4):315–409.

[66] Hayes RJ, Bennett S. Simple sample size calculation for cluster-randomized trials. Int J Epidemiol. 1999;28(2):319–26. 10.1093/ije/28.2.319 10342698

[67] Bot Factory^22^ Dialogue AI Platform Rapidly build your exclusive bot [https://www.emotibot.com/en/product/botfactory.html] Accessed on 20 May 2023.

[68] Toobert DJ, Hampson SE, Glasgow RE. The summary of diabetes self-care activities measure: results from 7 studies and a revised scale. Diabetes Care. 2000;23(7):943–50. 10.2337/diacare.23.7.943 10895844

[69] Craig CL, Marshall AL, Sjostrom M, Bauman AE, Booth ML, Ainsworth BE, Pratt M, Ekelund U, Yngve A, Sallis JF, et al. International physical activity questionnaire: 12-country reliability and validity. Med Sci Sports Exerc. 2003;35(8):1381–95. 10.1249/01.MSS.0000078924.61453.FB 12900694

[70] Kripalani S, Risser J, Gatti ME, Jacobson TA. Development and evaluation of the Adherence to Refills and Medications Scale (ARMS) among low-literacy patients with chronic disease. Value Health. 2009;12(1):118–23. 10.1111/j.1524-4733.2008.00400.x 19911444

[71] Spitzer RL, Kroenke K, Williams JB, Lowe B. A brief measure for assessing generalized anxiety disorder: the GAD-7. Arch Intern Med. 2006;166(10):1092–7. 10.1001/archinte.166.10.1092 16717171

[72] Kroenke K, Spitzer RL, Williams JB. The PHQ-9: validity of a brief depression severity measure. J Gen Intern Med. 2001;16(9):606–13. 10.1046/j.1525-1497.2001.016009606.x PMC1495268 11556941

[73] Warttig SL, Forshaw MJ, South J, White AK. New, normative, English-sample data for the Short Form Perceived Stress Scale (PSS-4). J Health Psychol. 2013;18(12):1617–28. 10.1177/1359105313508346 24155195

[74] Duong TV, Aringazina A, Kayupova G, Nurjanah, Pham TV, Pham KM, Truong TQ, Nguyen KT, Oo WM, Su TT et al: Development and validation of a new short-form health literacy instrument (HLS-SF12) for the general public in six asian countries. Health Lit Res Pract 2019, 3(2):e91-e102. 10.3928/24748307-20190225-01 PMC6607763 31294310

[75] Herdman M, Gudex C, Lloyd A, Janssen M, Kind P, Parkin D, Bonsel G, Badia X. Development and preliminary testing of the new five-level version of EQ-5D (EQ-5D-5L). Qual Life Res. 2011;20(10):1727–36. 10.1007/s11136-011-9903-x PMC3220807 21479777

