# Correction: Effect of Artificial Intelligence-based Health Education Accurately Linking System (AI-HEALS) for Type 2 diabetes self-management: protocol for a mixed-methods study

**DOI:** 10.1186/s12889-026-29452-0

**Published:** 2026-10-06

**Authors:** Yibo Wu, Hewei Min, Mingzi Li, Yuhui Shi, Aijuan Ma, Yumei Han, Yadi Gan, Xiaohui Guo, Xinying Sun

**Affiliations:** 1 Department of Social Medicine and Health Education, School of Public Health, Peking University, Beijing, China; 2 School of Nursing, Peking University, Beijing, China; 3 Beijing Center for Disease Control and Prevention, Beijing, China; 4 Beijing Medical Examination Center, Beijing, China; 5 Daxing District Center for Disease Control and Prevention of Beijing, Beijing, China; 6 Peking University First Hospital, Beijing, China

**Correction: BMC Public Health 23**,** 1325 (2023)**

**https://doi.org/10.1186/s12889-023-16066-z**

During the process of publishing the original article [1], the authors identified several reporting errors in the published protocol.

First, an error was identified in the sample size calculation and reporting. The intracluster correlation coefficient (ICC) was not changed and remained 0.002, as prespecified in the original approved protocol. The sample size calculation was based on HbA1c as the primary outcome, assuming a mean HbA1c of 7.13%, a standard deviation of 1.60%, an expected reduction in HbA1c to 6.50% after the intervention, a two-sided significance level of 0.05, 90% power, and an ICC of 0.002. Based on these assumptions, the required sample size was approximately 157 participants per group before allowing for loss to follow-up. After allowing for a 20% dropout rate, the final planned sample size was increased to 200 participants per group, yielding a total target sample size of 400 participants. The original article incorrectly stated that “a total of 664 patients in 4 communities are required to be recruited for this program.” In addition, the phrase “Based on our previous study” has been removed, as the sample size assumptions were prespecified in the original approved protocol rather than derived from a single previous study.

Second, the follow-up schedule for outcome assessment was incorrectly described in the published protocol. The original article listed assessment time points as 0 M, 1 M, 3 M, 6 M, 12 M, and 18 M. The correct outcome assessment time points are baseline (0 M), 3 M, 6 M, 12 M, and 18 M; there is no 1-month follow-up assessment.

Third, Table 1 has been corrected. Cloud data should be collected at 3 M only, and semistructured qualitative interviews should be conducted at 6 M only.

These revisions ensure that the published protocol accurately reflects the registered and implemented study procedures.

The original article [1] has been corrected.
